# Effects of High Pharmaceutical Concentrations in Domestic Wastewater on Membrane Bioreactor Treatment Systems: Performance and Microbial Community

**DOI:** 10.3390/membranes13070650

**Published:** 2023-07-06

**Authors:** Chengyue Li, Xin Du, Chuyi Huang, Zhenghua Zhang

**Affiliations:** 1 Membrane & Nanotechnology-Enabled Water Treatment Center, Tsinghua Shenzhen International Graduate School, Tsinghua University, Shenzhen 518055, China; 2 Guangdong Provincial Engineering Research Centre for Urban Water Recycling and Environmental Safety, Tsinghua Shenzhen International Graduate School, Tsinghua University, Shenzhen 518055, China; 3 School of Environment, Tsinghua University, Beijing 100084, China

**Keywords:** membrane bioreactor, pharmaceutical, microbial community, metabolic pathway, wastewater treatment

## Abstract

Despite pharmaceuticals being widely detected in water-bodies worldwide, what remain unclear are the effects of high pharmaceutical concentrations on the treatment efficiency of biological wastewater treatment processes, such as membrane bioreactor (MBR) systems. This study investigated the efficiency of MBR technology in the treatment of synthetic wastewater containing a mixture of five typical pharmaceuticals (ofloxacin, sulfamethoxazole, sulfamethylthiadiazole, carbamazepine and naproxen) with a total concentration of 500 µg/L. Both the control MBR (MBRc) without pharmaceutical dosing and the MBR operated with high influent pharmaceutical concentrations (MBRe) were operated under room temperature with the same hydraulic retention time of 11 h and the same sludge retention time of 30 d. The removal efficiency rates of total nitrogen and total phosphorus were 83.2% vs. 90.1% and 72.6% vs. 57.8% in the MBRc vs. MBRe systems, and both MBRs achieved >98% removal of organics for a 180-day period. The floc size decreased, and membrane fouling became more severe in the MBRe system. Microbial diversity increased in the MBRe system and the relative abundances of functional microbe differed between the two MBRs. Furthermore, the total relative abundances of genes involved in glycolysis, assimilating nitrate reduction and nitrification processes increased in the MBRe system, which could account for the higher organics and nitrogen removal performance. This work provides insights for MBR operation in wastewater treatment with high pharmaceutical concentrations.

## 1. Introduction

Membrane bioreactor (MBR) systems consist of bioreactors coupled with a membrane filtration unit [1]. MBR has been widely applied for the removal of nitrogen, phosphorus and micropollutants, as it has the advantages of high activated sludge concentrations, high volumetric loading rates, a small footprint, relatively low levels of sludge generation and the ability to achieve high performance levels by combining biological degradation and membrane filtration processes [2,3,4]. In recent years, pollutants originating from pharmaceuticals and personal care products (PPCPs) have been identified as key emerging micropollutants, which are widely detectable in natural water-bodies at concentrations in the ng/L to μg/L range, posing a threat to both environmental and human health [3]. The high concentration of PPCPs in natural water-bodies has been attributed to their widespread accessibility and high usage levels worldwide [5]. The distribution and concentration of PPCPs have been shown to be affected by both spatial and anthropogenic factors [6]. For example, it has been reported that a high concentration of sulfisoxazole (330.8 ng/L) was detected in a river located close to pharmaceutical manufacturer facilities in a coastal watershed of China [7]. Previous studies have reported micropollutant concentrations reaching 10^4^–10^5^ ng/L in wastewater treatment plant influent in Canada [8], while pharmaceutical concentrations ranging from 90–31,000 μg/L have been detected in pharmaceutical wastewater in Patancheru, near Hyderabad, India [9]. High concentrations of PPCPs have also been reported in landfill leachate. For example, 37 types of emerging contaminant were detected in landfill leachate (southern China), with concentrations in the range of 272–1780 μg/L [10].

The presence of high levels of PPCPs in wastewater can have serious toxic effects on aquatic ecosystems if not effectively removed. MBR systems can effectively remove PPCPs with the removal mechanism by MBR including volatilization, membrane retention, biodegradation and sludge adsorption [11,12]. Hena and Znad [13] summarized the average removal efficiency of MBR systems for various PPCPs, which ranged from 28% to 99.8%. However, most studies in this field have focused on the removal of PPCPs present at low concentrations, while few studies have investigated the removal of PPCPs at high concentrations [14]. In addition, MBR technology has been practically applied for the treatment of PPCP micropollutants, with reported removal efficiencies for the antibiotic ofloxacin and the non-steroidal anti-inflammatory naproxen ranging from 33.9% to 95.2% [15,16,17,18]. Sulfamethoxazole and sulfamethizole are commonly used sulfonamide antibiotics, and it has previously been reported that 20–92% of sulfamethoxazole can be eliminated by MBR treatment [19,20], while sulfamethizole was found to be the most recalcitrant sulfonamide with a negligible removal efficiency [21]. Similarly, the removal efficiency of the anti-epileptic drug carbamazepine ranged from negative to 23% [22]. The micropollutant removal efficiencies achieved using MBR systems have been shown to vary significantly due to different operational parameters, although the mechanisms of effect are unknown. Furthermore, it remains unclear the effect of high PPCPs concentrations on MBR wastewater treatment performance, in terms of microbial community dynamics, metabolic characteristics and membrane fouling.

Herein, the performance of MBR systems was investigated in the presence and absence of high pharmaceutical concentrations during the treatment of domestic wastewater, determining the treatment efficiency, effect on sludge properties and membrane fouling characteristics. Furthermore, 16S rRNA and metagenomic high-throughput sequencing were applied to analyze the effect of high pharmaceutical concentrations on the sludge microbial community characteristics. The aims of this study were to explain the mechanisms of pharmaceutical effect at high concentrations on water quality, microbial community structure and membrane fouling characteristics in the MBR system, providing theoretical guidance for the effective treatment of pharmaceutical contaminated wastewater using MBR systems.

## 2. Materials and Methods

### 2.1. Experimental Setup

**Figure 1:**
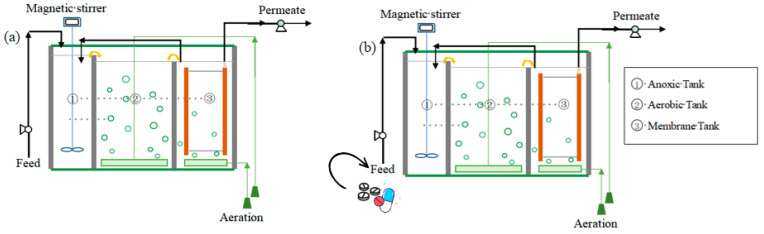
Schematic of the ceramic MBR system. (**a**) MBRc: control MBR; (**b**) MBRe: MBR operated with high influent pharmaceutical concentrations.

As shown in Figure 1, two laboratory-scale anoxic–aerobic ceramic MBRs were operated in parallel to investigate the effects of high pharmaceutical concentrations on domestic sewage treatment efficiency. The bioreactors were constructed of plexiglass and included an anoxic tank (2 L, DO = 0.5–1.0 mg/L), an aerobic zone (3 L, DO = 1.5–2.5 mg/L) and a membrane tank (2 L, DO = 1.5–2.5 mg/L). The anoxic–oxic–oxic membrane bioreactor has been proved to achieve high nutrients and pharmaceutical removal efficiency in our previous studies [11,12]. The active area of the flat sheet ceramic membrane (Al_2_O_3_, Meidensha Corp., Japan) was 0.043 m^2^, and the average pore size was 0.1 μm.

Both bioreactor systems were inoculated using seed sludge collected from a local wastewater treatment plant in Shenzhen (China). The anoxic sludge was injected into the anoxic chamber, while the aerobic sludge was fed into the aerobic and membrane chambers. To achieve stable performance, the MBRs were fed continuously with synthetic wastewater for a three-month period prior to use in experiments, with a permeate flux of 15 L/m^2^**^.^**h and a filtration/relaxation time ratio of 8/2 (minutes). Both MBRs were operated under the same environmental conditions at room temperature. Other operational conditions included a mixed liquor suspended solid (MLSS) concentration of 6–6.5 g/L, a hydraulic retention time (HRT) of 11 h, a sludge retention time (SRT) of 30 days and a recirculation ratio of 300%. The MBRs operated with or without the five micropollutants (ofloxacin, sulfamethoxazole, sulfamethylthiadiazole, carbamazepine and naproxen, with 100 µg/L each) added to the influent were labelled as MBRe and MBRc, respectively. A detailed description of the synthetic wastewater composition and the main physicochemical properties of the selected micropollutants are provided in Tables S1 and S2 (Supplementary Information). The trans-membrane pressure (TMP) of the membrane module was monitored continuously in each MBR, and when the normalized TMP (ΔTMP = total TMP − intrinsic membrane TMP) was above 30 kPa, physical and chemical cleaning methods were implemented to ensure the continuous operation at the constant membrane flux of 15 L/m^2^**^.^**h [23,24].

### 2.2. Analytical Methods

Samples were collected for dissolved organic carbon (DOC), total nitrogen (TN) and total phosphorus (TP) quantification from the influent, effluent and the supernatant of the anoxic and aerobic tanks of each MBR system. Briefly, the DOC concentrations were measured using a TOC analyzer (TOC-VCSH, Shimadzu, Japan). TP and TN concentrations were determined using the ascorbic acid reduction method and the spectrophotometric screening method after persulfate digestion, respectively. Protein and polysaccharide concentrations are the main components of soluble microbial products (SMP) and extracellular polymeric substances (EPS), and they were determined using the Lowry and phenol/H_2_SO_4_ methods, respectively [25]. The average floc size was measured using a particle size analyzer (Master-sizer MS3000, Malvern, UK) with data expressed as a volume equivalent diameter (Dx 50). The excitation–emission matrix (EEM) was obtained using a fluorescence spectrometer (F-700, Hitachi, Japan), and the fluorescence regional integration (FRI) method was applied to compare the distribution of dissolved organic matter (DOM) in samples [26]. Finally, a liquid chromatography-ultraviolet (LC-UV) system (LC-20AT, Shimadzu, Japan) was used to determine the molecular weight (MW) distribution of supernatant samples.

### 2.3. Analysis of Microbial Taxa and Functional Genes

Samples were collected from the anoxic tank, aerobic tank and membrane tank from each MBR for microbial community analysis at the 1st, 100th and 180th days (Magigene Biotechnology Co. Ltd., Guangzhou, China). A detailed description of the procedure used for microbial community analysis has been reported by Ren et al. [24] and is provided in the Supplementary Information (Text S1). Metagenomic analysis was conducted using the Illumina NovaSeq platform. Quality control was performed using Trimmomactic (v.0.32), with the clean reads then assembled using MEGAHIT (v.1.0.6) (k-min 35, k-max 95, k-step 20) to obtain assembly contigs with a minimum length of 500 bp [27,28]. The open reading frames (ORFs) were predicted using Prodigal (v.2.6.3) and clustered using Linclust to acquire gene catalogues [29,30,31]. Finally, the functional genes were annotated against the Kyoto Encyclopedia of Genes and Genomes (KEGG) database based on an E-value threshold of 10^−5^ [32].

## 3. Results and Discussion

### 3.1. Effluent Water Quality

**Figure 2:**
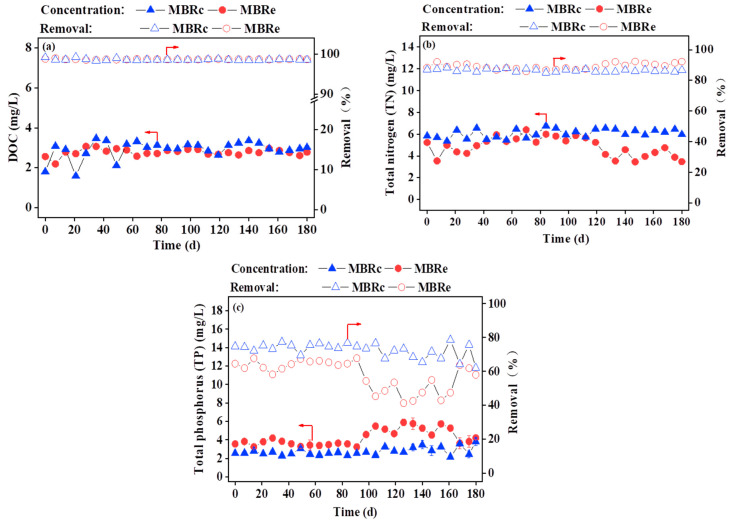
MBR effluent concentration and removal efficiency of (**a**) DOC, (**b**) TN and (**c**) TP in the presence and absence of high pharmaceutical concentrations for the control MBR (MBRc) and experimental MBR (MBRe) systems.

The concentrations of DOC and TN in the effluent of each MBR system were monitored at weekly intervals for 180 days, as presented in Figure 2. Both MBRs were able to effectively remove >98% of organics, with average DOC concentrations in the MBRc and MBRe effluents of 2.92 and 2.78 mg/L, respectively (Figure 2a). High influent pharmaceutical compound concentrations had no significant adverse impact on the DOC removal efficiency, which may have remained consistently high due to DOC biodegradation by activated sludge [23]. The obtained EEM spectra (Figure S1) were divided into five regions including protein-like (regions I and II, Ex/Em = 220–250/280–330 nm, and Ex/Em = 220–250/330–380 nm), fulvic-like (region III, Ex/Em = 220–250/380–480 nm), SMP-like (region IV, Ex/Em = 250–440/280–380 nm) and humic-like (region V, Ex/Em = 250–400/380–540 nm) substances [26]. According to the fluorescence regional integration method presented in Table 1, the intensities of effluent fluorescence decreased in both MBRs with time, although the addition of pharmaceuticals appears to have a negative effect on the removal of fluorescent substances.

The average TN concentration in the effluent of both MBRs met the requirements of the Chinese discharge standard, although the TN removal efficiency was slightly higher in the MBRe system (90.1%) than the MBRc system (83.2%) (Figure 2b). However, the average TP concentrations in the MBRc and MBRe effluents were 2.74 ± 0.42 and 4.22 ± 0.85 mg/L, respectively (Figure 2c), showing that the MBRe system achieved a poorer TP removal efficiency than the MBRc system. It has previously been reported that higher pharmaceutical concentrations may have a negative impact on polyphosphate-accumulating organisms (PAOs), affecting the TP removal efficiency [33]. A detailed discussion of the microbial community compositions responsible for nutrient removal is presented in the following section.

### 3.2. Mixed Liquor Properties

**Figure 3:**
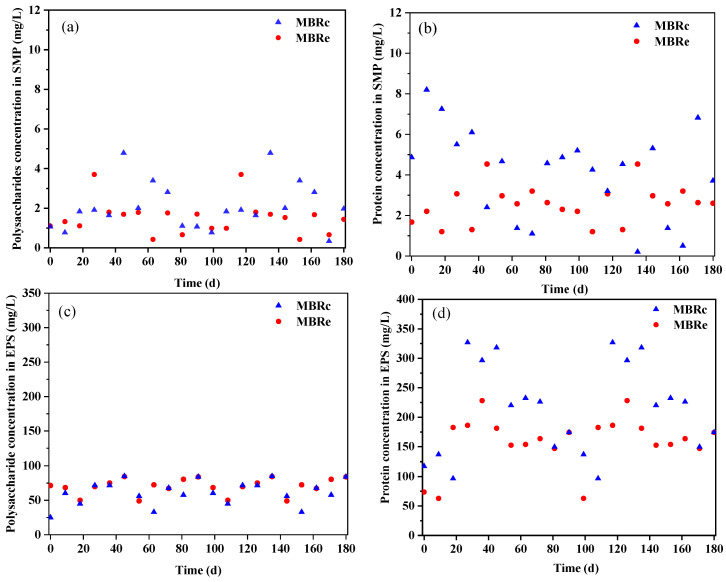
Polysaccharide and protein concentrations in the SMP (**a**,**b**) and EPS (**c**,**d**) of the control MBR (MBRc) and experimental MBR (MBRe) systems.

The polysaccharide and protein compositions of SMP and EPS in each MBR system are shown in Figure 3. The average SMP polysaccharide and protein concentrations in the aerobic tank of the MBRe system were 1.52 and 2.57 mg/L, showing a reduction compared to the MBRc system at 2.09 and 4.10 mg/L, respectively. However, the average EPS polysaccharide concentration in the MBRe system was 71.32 mg/L, while in the MBRc, it was only 65.11 mg/L. Conversely, the EPS protein concentration in the MBRc system reached 213.82 mg/L, which was significantly higher than in the MBRe system, reaching only 164.26 mg/L. In summary, SMP and EPS secretion can be affected when the microbial community is exposed to high concentrations of pharmaceutical compounds.

**Figure 4:**
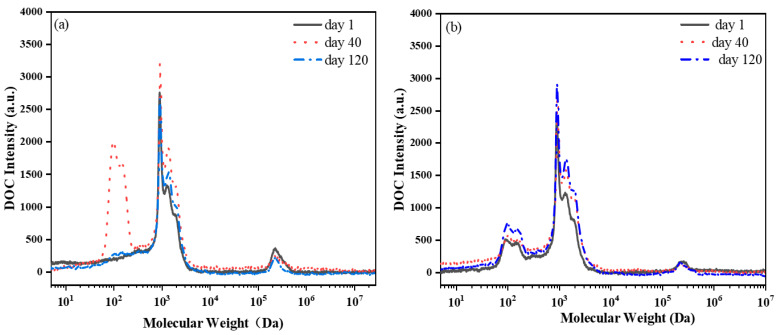
The selected molecular weight of SMP samples from aerobic tank of the (**a**) control MBR (MBRc) and (**b**) experimental MBR (MBRe) systems.

By comparing the molecular weight distribution of SMP samples from the aerobic tank of each MBR system (Figure 4), a slight increase in substances with weights of 100 Da was observed in the MBRe system, as compared to the MBRc, except for the MBRc on the 40th day, which exhibited a large increase in DOC intensity. However, a decrease in DOC intensity was also observed for substances in the size range of 10^5^–10^6^ Da in the MBRe system, compared to the MBRc. The differences in changes in SMP molecular weight distribution may be associated with the variation in microbial activity and metabolic function after the addition of pharmaceuticals [11,12].

**Figure 5:**
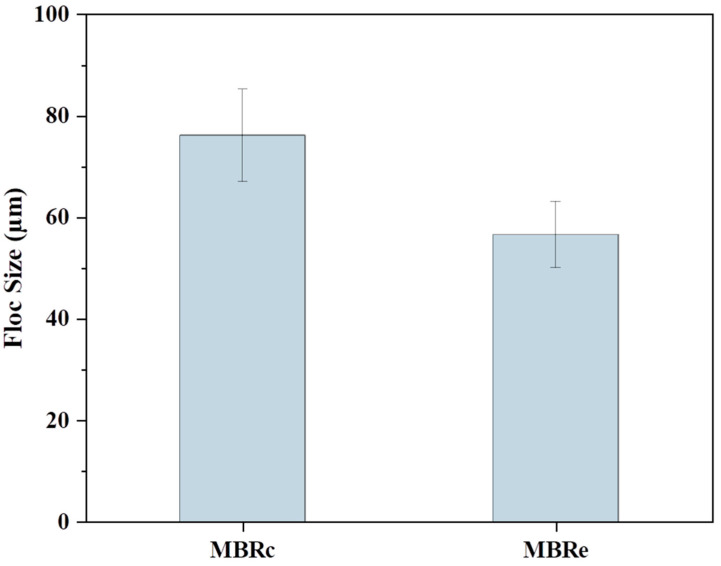
Floc size in the anoxic zones of the control MBR (MBRc) and experimental MBR (MBRe) systems.

High pharmaceutical concentrations not only affected the SMP and EPS compositions, but also altered the floc size. As shown in Figure 5, the average floc size varied in the aerobic tank of each MBR. In the MBRc system, the average floc size in the aerobic tank was 76.15 µm. However, the average floc size in the aerobic tank of the MBRe system was 56.58 µm. The observed reduction in floc size due to the addition of pharmaceuticals is in agreement with a previous study showing that floc size decreased from 389 µm to 202 µm following the addition of fluoroquinolone antibiotics into a MBR system [34]. The possible reason could be the death of bacteria cells and deflocculation of activated sludge when exposed to toxic pharmaceuticals [35].

### 3.3. Microbial Community Characterization

#### 3.3.1. Alpha Biodiversity Analysis

The microbial community composition has a major impact on the stability of activated sludge processes and the ability of microbes to transform nutrients [36]. To study the effect of high pharmaceutical concentrations on the microbial community structure and metabolic characteristics, samples were collected from the anoxic and aerobic tanks for 16S rRNA high-throughput sequencing analysis at the start (day 1), middle (day 100) and end (day 180) of the experimental period.

The sequencing of bacterial 16S rRNA genes generated 64,091 to 75,051 and 64,395 to 76,220 active sequences in total from the samples obtained from the anoxic and aerobic tanks of the MBRc and MBRe systems, respectively (Table 2). The number of OTUs identified using the UPARSE method (with a 97% threshold value) in the anoxic tank of the MBRe system was higher (1470 on the 180th day) than in the MBRc anaerobic tank (1317 on the 180th day). A similar trend was observed in the number of OTUs in the aerobic tanks of each MBR system, increasing from 1118 to 1290 in the MBRc and from 1190 to 1431 in the MBRe. Chao, Shannon and Simpson indices were used to assess the richness, diversity and evenness of microbial communities. An increase in microbial richness was observed following the addition of high pharmaceutical concentrations, with Chao index values of 1291–1318 and 1417–1432 in the MBRc and MBRe systems, respectively. On the 180th day, the Shannon index value was higher in the MBRe (7.12–7.21) than in the MBRc (5.95–5.97), indicating an increase in bacterial community diversity after the addition of pharmaceuticals, with this result supported by the Simpson index values (0.018–0.024 for MBRc vs. 0.059–0.060 for MBRe). Overall, these findings are in agreement with a previous study showing that the addition of PPCPs at concentrations below 2 mg/L could increase microbial diversity and promote microbial growth [14].

#### 3.3.2. Microbial Taxonomic Analysis

**Figure 6:**
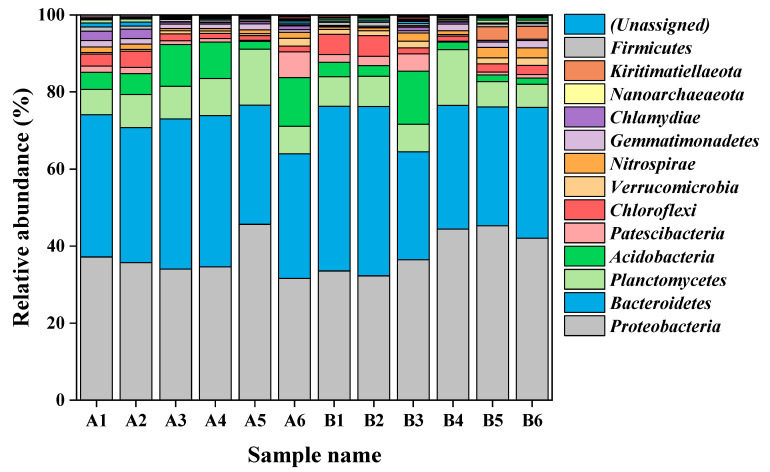
Relative abundance of the 20 most dominant species at the phylum level (A1: MBRc day 1/anoxic; A2: MBRc day 1/oxic; A3: MBRc day 100/anoxic; A4: MBRc day 100/oxic; A5: MBRc day 180/anoxic; A6: MBRc day 180/oxic; B1: MBRe day 1/anoxic; B2: MBRe day 1/oxic; B3: MBRe day 100/anoxic; B4: MBRe day 100/oxic; B5: MBRe day 180/anoxic; B6: MBRe day 180/oxic).

The microbial communities were investigated at the phylum and genus levels to compare the effect of additional pharmaceuticals on wastewater treatment in each MBR. As shown in Figure 6, Proteobacteria and Bacteroidetes were the dominant phyla in both MBR systems, which was in accordance with results previously reported in the literature [37,38]. Compared to the initial stage of operation (day 1), Proteobacteria were eventually enriched in both the MBRc and MBRe systems, resulting in Proteobacteria being dominant in the anoxic and aerobic tanks of both MBRs by the final stage of operation (day 180). Therefore, the addition of high pharmaceutical concentrations had no significant effect on Proteobacteria within the MBR systems. Proteobacteria have been reported to play an important role in the removal of antimicrobial and anti-inflammatory substances, and previous studies have shown that PPCPs can promote the dominance of Proteobacteria and Bacteroidetes [39]. Acidobacteria are closely associated with the degradation of organic compounds [40], with the relative abundance of Acidobacteria increasing initially in both MBR systems, before finally decreasing. In addition, by the 180th day, the relative abundances of Nitrospirae and Kiritimatiellaeota had increased in the MBRe system compared to the MBRc system, which was associated with nitrogen removal [41,42]. However, following the addition of pharmaceuticals, the relative abundance of Planctomycetes decreased in the anoxic and aerobic tanks by 16% and 54% compared to the MBRc system, which may be due to some pharmaceuticals, such as carbamazepine, being toxic to these bacteria [39].

**Figure 7:**
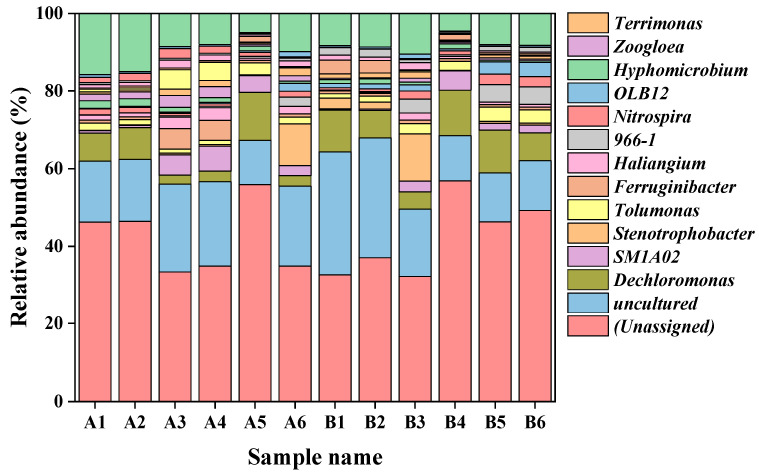
Relative abundance of the 20 most dominant species at the genus level (A1: MBRc day 1/anoxic; A2: MBRc day 1/oxic; A3: MBRc day 100/anoxic; A4: MBRc day 100/oxic; A5: MBRc day 180/anoxic; A6: MBRc day 180/oxic; B1: MBRe day 1/anoxic; B2: MBRe day 1/oxic; B3: MBRe day 100/anoxic; B4: MBRe day 100/oxic; B5: MBRe day 180/anoxic; B6: MBRe day 180/oxic).

At the genus level (Figure 7), after 180 days, the addition of pharmaceuticals increased the abundance of genera such as *Tolumonas*, *Haliangium*, *Nitrospira* and *Terrimonas*, as compared to the MBRc system. *Nitrospira* are classed as nitrifying bacteria, while *Haliangium* and *Terrimonas* are responsible for denitrification [24,43]. The enrichment of these bacterial genera contributed to the nitrogen removal performance of the MBRe system. However, compared with the MBRc system, a decrease was observed in the relative abundance of *Dechloromonas* in the MBRe system in the final stage of operation (day 180), which may explain the poor phosphate removal efficiency in the MBRe system treating high influent pharmaceutical concentrations as these are denitrifying phosphate-accumulating organisms (DPAOs) [44]. *Tolumonas* has the capability to degrade complex organic contaminants and PPCPs, while *Terrimonas* have been reported to metabolize recalcitrant aromatic hydrocarbons [45,46,47]. Once complex PPCP structures are metabolized by functional bacteria, the reduced PPCPs can be further transformed into persistent PPCP pollutants which are less toxic (or non-toxic) to the bacterial community [48,49]. Overall, the observed microbial community dynamics could be due to microbial self-defense mechanisms responding to the stress of pharmaceutical exposure [14].

The addition of high pharmaceutical concentrations could have an impact on the metabolic characteristic of microbial communities. Therefore, the carbon and nitrogen metabolic pathways and variations in functional genes were compared in each MBR system, to determine the effect of high pharmaceutical concentrations on the MBR performance.

#### 3.3.3. Potential Pathways for Nitrogen and Carbohydrate Transformation in Each MBR

**Figure 8:**
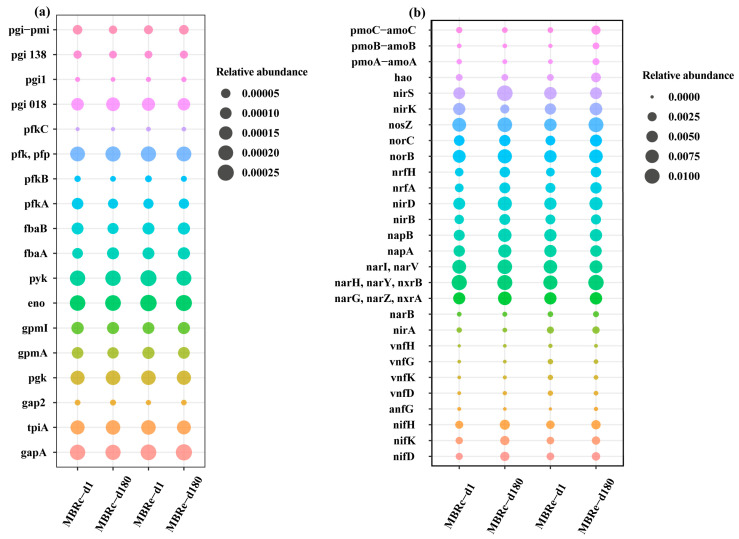
Relative abundances of functional genes involved in (**a**) carbon metabolism by glycolysis and (**b**) nitrogen metabolism in the MBRc and MBRe systems.

In this study, two fundamental metabolic pathways, glycolysis and nitrogen metabolism processes were studied to determine the effect of high pharmaceutical concentrations on microbial function and activity. Glucose utilization plays a vital role in energy metabolism [50], and therefore, in order to assess the influence of high pharmaceutical concentrations on microbial carbon metabolism, the variations in functional genes involved in glycolysis were analyzed in each MBR. As shown in Figure S2 and Figure 8a, after 180 days of operation, the relative abundances of pfk, a gene relating to the initial part of the glycolysis process, were 0.0000144% and 0.000066% in the MBRc and MBRe systems, respectively, exhibiting a significant increase following the addition of pharmaceuticals. In addition, the total relative abundances of key glycolysis process genes in the MBRc were 0.193% (day 1) and 0.194% (day 180), while in the MBRe system, they were 0.205% (day 1) and 0.198% (day 180). Therefore, after the addition of pharmaceuticals, the total relative abundance of genes involved in the glycolysis process were slightly increased, implying enhanced microbial activity [51] and resulting in higher organics removal in the MBRe system. This result is consistent with the lower DOC concentration detected in the effluent of the MBRe system, as compared to the MBRc system (Figure 2).

To further understand the role of microorganisms in nitrogen conversion, the relative abundances of genes encoding key enzymes involved in nitrogen metabolism were determined. As shown in Figure S3 and Figure 8b, compared to the MBRc system on the 180th day of operation the total relative abundances of functional genes in the MBRe involved in nitrogen conversation processes: nitrogen fixation, dissimilatory nitrate reduction and ammonification reduced by 24.66%, 14.43%, 3.877% and 12.51%, respectively, while genes associated with assimilatory nitrate reduction and nitrification increased by 742.17% and 359.37%, respectively. The high relative abundance of nitrification genes was consistent with the observed increasing trend in total nitrogen removal efficiency in the MBRe system, since nitrification was the rate-limiting step of the biological nitrogen removal [52].

### 3.4. Membrane Fouling

**Figure 9:**
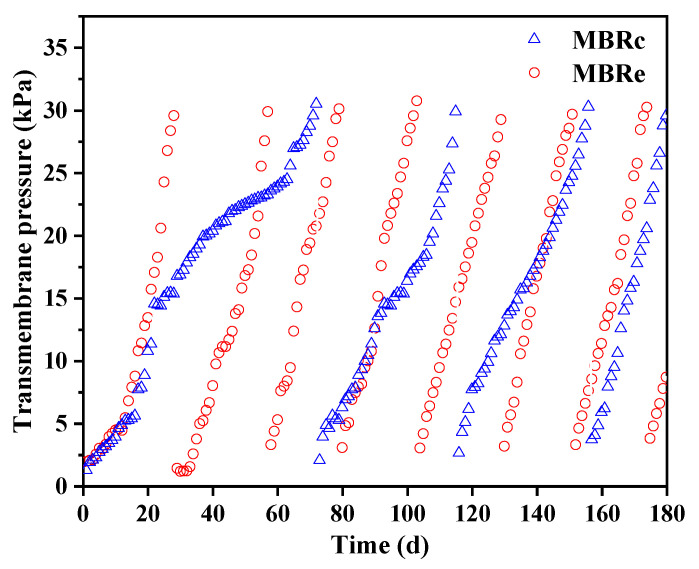
Transmembrane pressure in the control and experimental MBR systems: MBRc = without pharmaceuticals; MBRe = with the addition of high pharmaceutical concentrations.

The presence of PPCPs is also a major cause of membrane fouling, which severely limits the operational performance of MBR systems [13], and therefore, the effect of high pharmaceutical concentrations on membrane fouling was investigated. TMP was continuously monitored as shown in Figure 9. Compared to the MBRc system, the MBRe exhibited rapid membrane fouling, requiring approximately 28 days for the ΔTMP to increase to 30 kPa in the MBRe, while around 72 days were required in the MBRc. During the first 13 days of operation, there was slight difference observed in the ΔTMP values of each MBR system. Subsequently, the membrane fouling rate in the MBRc system remained around 1.11 kPa/d from day 14 to day 22, and then it reduced to 0.26 kPa/d until after the 64th day, when the membrane fouling rate increased to 0.62 kPa/d. In contrast, the membrane fouling rate in the MBRe was faster overall, reaching around 1.67 kPa/d from day 14 to day 28. The acceleration of membrane fouling caused by high pharmaceutical concentrations was consistent with a previous study [53], reporting that the addition of 90 μg/L carbamazepine to an MBR system increased the TMP after 1 day, which was attributed to the change in EPS concentrations and sludge deflocculation caused by activated sludge exposure to toxic chemicals. In this study, the observed reduction in floc size in the MBRe (mean value: 56.58 µm for MBRe vs. 76.15 µm for MBRc) could also aggravate membrane fouling, which is supported by previous studies showing that microbial physiological and ecological adjustments for self-protection against high levels of PPCPs are an important cause of membrane fouling [13]. Also, the severe membrane fouling in the MBRe might be due to the high polysaccharide concentration in EPS (mean value: 71.32 mg/L for MBRe vs. 65.11 mg/L for MBRc), referring to the previous studies which mentioned that the polysaccharide could contribute more significantly to the thickness of biofilm compared to protein [54,55].

## 4. Conclusions

In this study, a mixture of five typical pharmaceuticals (ofloxacin, sulfamethoxazole, sulfamethoxazole, carbamazepine, naproxen) with a total concentration of 500 µg/L, was added to the influent of an experimental membrane bioreactor system. The effluent quality, sludge properties and degree of membrane fouling were assessed in each MBR system. In addition, the microbial community dynamics and metabolic characteristics were compared in the presence and absence of high pharmaceutical concentrations, in order to determine the effect of high pharmaceutical concentrations on the treatment efficiency of domestic sewage by a ceramic membrane bioreactor system. The conclusions are as follows:

(1) Both MBR systems could remove organics effectively, although the TN removal efficiency was higher in the MBRe system (approximately 90%) than in the MBRc system (83.20%). However, the TP removal efficiency in the MBRe was 19.70% lower than in the MBRc. In addition, membrane fouling became severe, and the floc size decreased after the addition of pharmaceuticals.
(2) The addition of high pharmaceutical concentrations had no significant effect on the relative abundance of Proteobacteria, while the relative abundances of Nitrospirae and Kiritimatiellaeota increased in the MBRe system compared to the MBRc system. However, after 180 days of operation, the relative abundance of *Dechloromonas* decreased in the MBRe compared to the MBRc, which may contribute to the poor phosphate removal efficiency of the MBRe system.
(3) After the addition of high pharmaceutical concentrations (at 180 days), the total relative abundance of genes involved in glycolysis, assimilatory nitrate reduction and nitrification processes increased, which could account for the higher organics and nitrogen removal in the MBRe compared to the MBRc. This work could reveal the effects of high pharmaceutical concentrations on the treatment efficiency of MBR and provide suggestions for MBR operation in practical applications.

## Figures and Tables

**Table 1:** Partition of intensity in each region of the fluorescence spectra for the control MBR (MBRc) and experimental MBR (MBRe) systems.

Sample	I	II	III	IV	V
MBRc day 10	1.38 × 10^7^	2.91 × 10^7^	1.90 × 10^7^	1.75 × 10^7^	1.16 × 10^7^
MBRe day 10	1.37 × 10^7^	3.07 × 10^7^	2.24 × 10^7^	1.85 × 10^7^	1.40 × 10^7^
MBRc day 100	1.25 × 10^7^	2.42 × 10^7^	1.85 × 10^7^	1.62 × 10^7^	1.16 × 10^7^
MBRe day 100	8.85 × 10^6^	2.06 × 10^7^	1.64 × 10^7^	1.52 × 10^7^	1.14 × 10^7^
MBRc day 180	4.89 × 10^6^	1.22 × 10^7^	1.28 × 10^7^	1.39 × 10^7^	1.10 × 10^7^
MBRe day 180	9.45 × 10^6^	2.18 × 10^7^	1.63 × 10^7^	1.75 × 10^7^	1.26 × 10^7^

**Table 2:** Bacterial 16S rRNA gene sequencing results and alpha diversity indices for the microbial taxa in the control MBR (MBRc) and experimental MBR (MBRe) systems in different operational periods.

Sludge Samples Description	Serial No.	Reads	OTUs	Chao	Shannon Index	Simpson Index
MBRc d 1/anoxic	A1	70,065	1227	1228	7.18	0.030
MBRc d 1/oxic	A2	64,091	1188	1189	7.11	0.032
MBRc d 100/anoxic	A3	75,051	1430	1431	6.93	0.023
MBRc d 100/oxic	A4	73,113	1359	1360	6.91	0.024
MBRc d 180/anoxic	A5	74,864	1317	1318	5.97	0.059
MBRc d 180/oxic	A6	74,001	1290	1291	5.95	0.060
MBRe d 1/anoxic	B1	64,931	1193	1194	6.42	0.062
MBRe d 1/oxic	B2	64,395	1198	1199	6.64	0.051
MBRe d 100/anoxic	B3	76,220	1540	1541	7.02	0.031
MBRe d 100/oxic	B4	65,816	1374	1375	6.93	0.033
MBRe d 180/anoxic	B5	74,241	1470	1471	7.12	0.024
MBRe d 180/oxic	B6	71,799	1431	1432	7.21	0.018

## Data Availability

All data are available in the main text or Supplementary Materials.

## Supplementary Materials

The following supporting information can be downloaded at: https://www.mdpi.com/article/10.3390/membranes13070650/s1, Text S1. Microbial community analysis. Table S1. Composition and characteristics of synthetic wastewater used in this study. Table S2. Physicochemical properties of the selected micropollutants. Figure S1. Fluorescence spectra of effluent for the control (MBRc) and experimental MBRs (MBRe) (a: 10th day of MBRc, b: 10th day of MBRe, c: 100th day of MBRc, d: 100th day of MBRe, e: 180th day of MBRc, f: 180th day of MBRe). Figure S2. Schematic of glycolysis pathway. Figure S3. Schematic of nitrogen metabolism.
