# Supplementary material for: Effects of High Pharmaceutical Concentrations in Domestic Wastewater on Membrane Bioreactor Treatment Systems: Performance and Microbial Community
Source: Membranes (Basel). 2023 Jul 6;13(7):650. doi: 10.3390/membranes13070650 (PMC10383461; doi:10.3390/membranes13070650)
Supplement: Supplementary file 1 [file membranes-13-00650-s001.zip › membranes-2458725-supplementary.pdf]

# Supplementary Information

# Effects of high pharmaceutical concentrations in domestic wastewater on membrane bioreactor treatment systems: Performance and microbial community

Chengyue Li [1,2,3,†], Xin Du [1,2,3,†], Chuyi Huang [1,2,3] and Zhenghua Zhang [1,2,3,*]

[1]Membrane & Nanotechnology-Enabled Water Treatment Center, Institute of Environmental Engineering & Nano-Technology, Tsinghua Shenzhen International Graduate School, Tsinghua University, Shenzhen 518055, Guangdong, China

[2]Guangdong Provincial Engineering Research Centre for Urban Water Recycling and Environmental Safety, Tsinghua Shenzhen International Graduate School, Tsinghua University, Shenzhen, 518055, Guangdong, China

[3]School of Environment, Tsinghua University, Beijing 100084, China

**Membranes**

Submitted on June 2023

[†]These authors contributed equally to this work.

[*]Correspondence: zhenghua.zhang@sz.tsinghua.edu.cn

**Text S1. Microbial community analysis**

Sludge samples collected from anoxic and aerobic tanks were centrifuged (4000 rpm, 8 min) before DNA extraction with PowerSoil DNA Isolation Kit (MOBIO, USA). The amplification of extracted DNA was conducted with a BioRad PCR (S1000, USA) using 515F and 806R primers. The NEBNext® Ultra™ DNA Library Prep Kit for Illumina® was utilized to generate sequencing libraries. Finally, PCR products were sequenced on an Illumina Hiseq2500 platform to generate 250 bp paired end reads.

The microbial community analysis was performed by following the procedures described in previous literature [1]. Initially, quality control of the paired-end raw reads was carried out using the Trimmomatic software (V0.33) followed by merging of the paired-end clean reads using the FLASH software (V1.2.11). Then, analysis of the obtained sequences was conducted by using the Unsearch software (V8.0.1517), and all the sequences with above 90% similarity were assigned to the same operational taxonomic units (OTU). Usearch software and UCHIME de novo algorithm were applied to remove singleton OTU and chimera, respectively. Ribosomal Database Project (RDP) classifier algorithm and the assign_taxonomy.py script in QIIME were used to annotate the sequences. The taxonomic information for each sequence was annotated using the Silva (16S) database. The number of observed species and Chao, Shannon and Simpson indices were determined using QIIME (V1.9.1) to asses microbial diversity and richness.

**Table S1.** Composition and characteristics of synthetic wastewater used in this study.

| Ingredients | Concentration | Parameters | Concentration |
|---|---|---|---|
| Glucose | 180 mg/L | TN (Total nitrogen) | 40-50 mg/L |
| Sodium acetate | 320 mg/L | TP (Total phosphorus) | 10 mg/L |
| Peptone | 15 mg/L | $NH_4^+$-N (Ammonia-N) | 25-30 mg/L |
| Malt extract | 15 mg/L | TOC (Total organic carbon) | 200-220 mg/L |
| Ammonium chloride | 140 mg/L | $COD_{cr}$ (Chemical oxygen demand) | 400-450 mg/L |
| $KH_2PO_4$ | 35 mg/L | $BOD_5$ (Biological oxygen demand) | 200-250 mg/L |
| $MgSO_4 \cdot 7H_2O$ | 300 mg/L | | |
| $FeSO_4 \cdot 7H_2O$ | 5 mg/L | | |

**Table S2.** Physicochemical properties of the selected micropollutants.

| Drugs | CAS No. | MW （g/mol） | $pK_a$ | $LogK_{ow}$ |
|---|---|---|---|---|
| Ofloxacin | 82419-36-1 | 361.37 | 7.90 | -0.39 |
| Sulfamethoxazole | 144-82-1 | 270.3 | 5.45 | 0.54 |
| Sulfamethizole | 723-46-6 | 253.3 | 5.60 | -0.22 |
| Carbamazepine | 298-46-4 | 236.3 | 13.94 | 1.89 |
| Naproxen | 22204-53-1 | 230.26 | 4.39 | 0.73 |

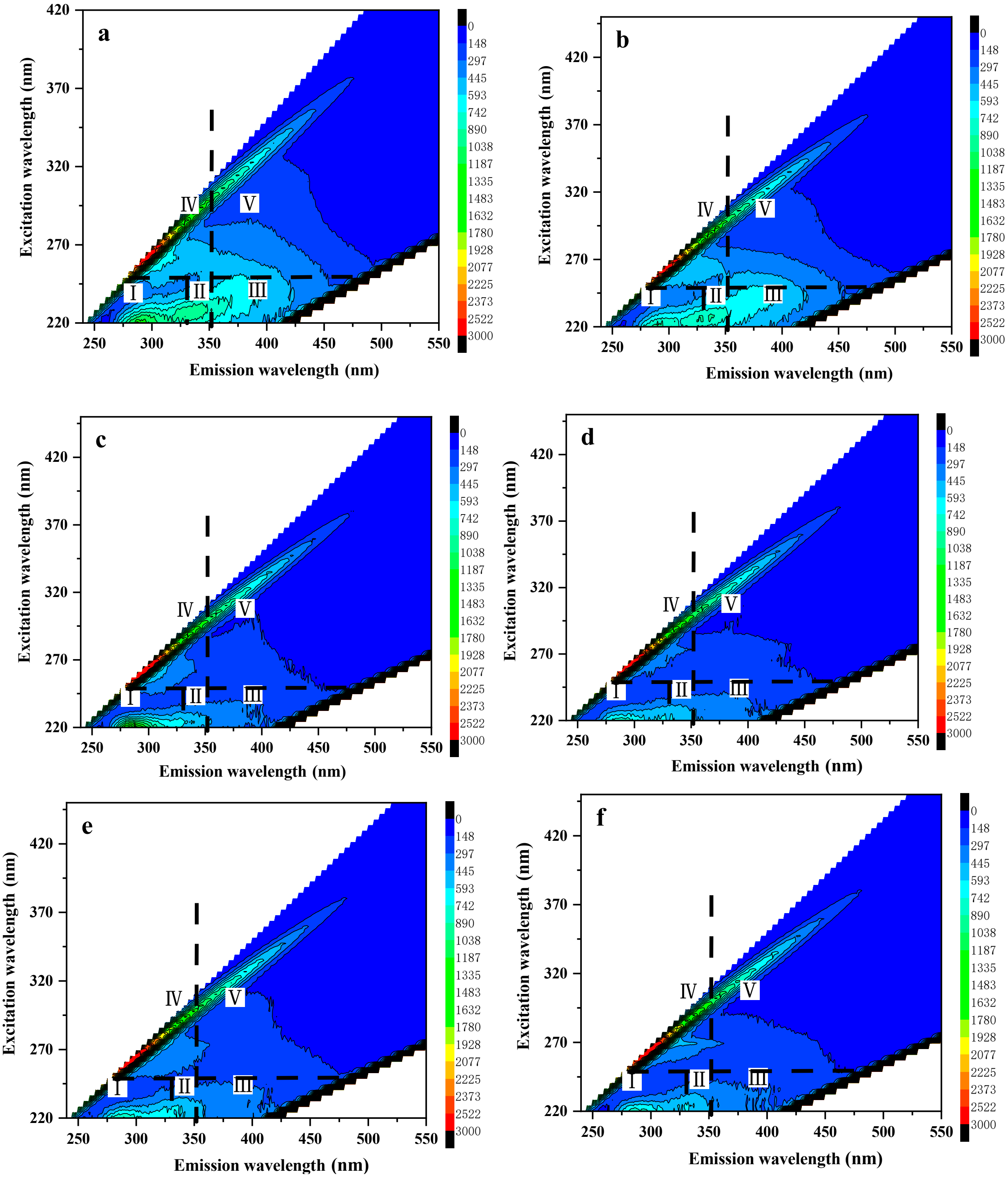


**Figure S1.** Fluorescence spectra of effluent for the control (MBRc) and experimental MBRs (MBRe) (a: 10th day of MBRc, b: 10th day of MBRe, c: 100th day of MBRc, d: 100th day of MBRe, e: 180th day of MBRc, f: 180th day of MBRe).

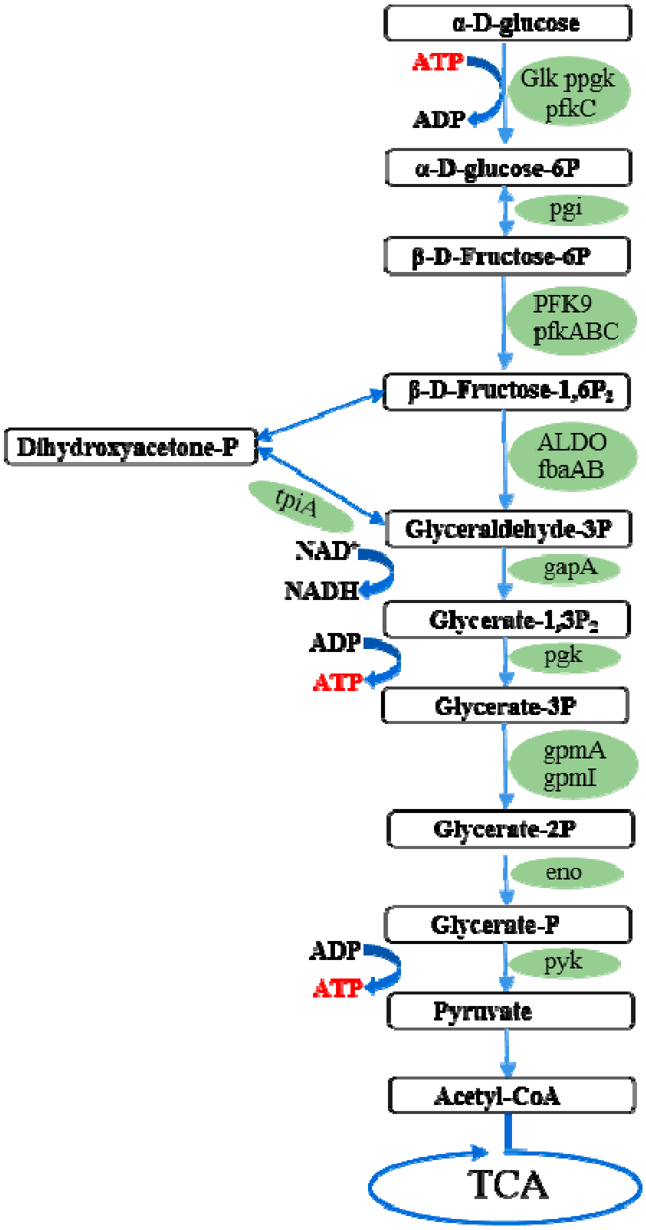


**Figure S2.** Schematic of glycolysis pathway.

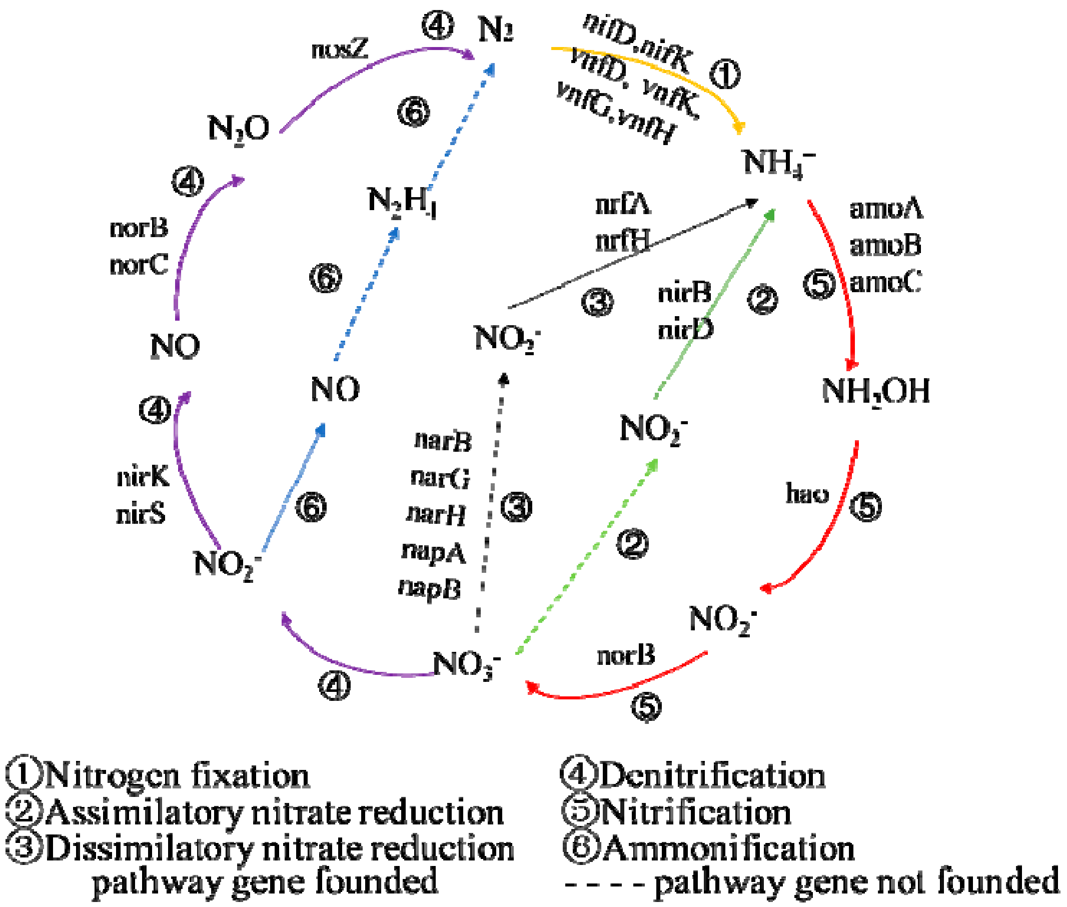


**Figure S3.** Schematic of nitrogen metabolism.

**Reference**

[1] B. Ren, C. Li, X. Zhang, Z. Zhang, Fe(II)-dosed ceramic membrane bioreactor for wastewater treatment: Nutrient removal, microbial community and membrane fouling analysis, Sci. Total Environ., 664 (2019) 116-126.
